# A Systematic Review of the Modifying Effect of Anaesthetic Drugs on Metastasis in Animal Models for Cancer

**DOI:** 10.1371/journal.pone.0156152

**Published:** 2016-05-26

**Authors:** Carlijn R. Hooijmans, Florentine J. Geessink, Merel Ritskes-Hoitinga, Gert Jan Scheffer

**Affiliations:** 1 Department of SYstematic Review Centre for Laboratory animal Experimentation (SYRCLE), Radboud University Medical Centre, 6500 HB, Nijmegen, the Netherlands; 2 Department of Anesthesiology, Pain and Palliative Medicine, Radboud University Medical Centre, 6500 HB, Nijmegen, the Netherlands; Imperial College London, Chelsea & Westminster Hospital, UNITED KINGDOM

## Abstract

**Background:**

Distant metastasis or local recurrence after primary tumour resection remain a major clinical problem. The anaesthetic technique used during oncologic surgery is suggested to influence the metastatic process. While awaiting the results of ongoing randomised controlled trials (RCTs), we have analyzed the evidence regarding the influence of anaesthetic drugs on experimental tumour metastasis in animal studies.

**Methods:**

PubMed and Embase were searched until April 21^st^, 2015. Studies were included in the systematic review when they 1) assessed the effect of an anaesthetic drug used in clinical practice on the number or incidence of metastasis in animal models with experimental cancer, 2) included an appropriate control group, and 3) presented unique data.

**Results:**

20 studies met the inclusion criteria (published between 1958–2010). Data on number of metastases could be retrieved from 17 studies. These studies described 41 independent comparisons, 33 of which could be included in the meta-analysis (MA). The incidence of metastases was studied in 3 unique papers. From these 3 papers, data on 7 independent comparisons could be extracted and included in the MA. Locally administered local anaesthetics appear to decrease the number of metastases (SMD -6.15 [-8.42; -3.88]), whereas general anaesthetics (RD: 0.136 [0.045, 0.226]), and more specifically volatile anaesthetics (SMD 0.54 [0.24; 0.84]), appear to increase the number and risk of metastases in animal models for cancer.

**Conclusions:**

Anaesthetics influence the number and incidence of metastases in experimental cancer models. Although more high quality experimental research is necessary, based on the currently available evidence from animal studies, there is no indication to suggest that locally administered local anaesthetics are harmful during surgery in cancer patients. Volatile anaesthetics, however, might increase metastasis in animal models and clinical trials investigating this possibly harmful effect should receive priority. The results of our systematic review in animal studies are broadly consistent with clinical reports that anaesthetic technique does seem to affect the tumour metastasis process.

## Introduction

Despite the progress made in cancer treatment, distant metastasis or local recurrence after primary tumour resection remain a major clinical problem. As a consequence, a lot of research concentrates on exploring factors that might influence the metastatic process. Some perioperative factors for example, appear to directly affect cancer cells, and can also have an impact on cell-mediated immunity. This way, these factors can promote the complex process of metastasis (summarized in [1]). One of the factors that has been suggested to influence tumour recurrence or metastasis is the anaesthetic technique applied during oncologic surgery. Inhalation anaesthetics have been suggested to increase cancer recurrence [2], whereas regional anaesthesia, in contrast, may decrease distant metastasis [3]. The effect of intravenously administered anaesthetics on tumour metastasis appears to vary with the type of drug [4]. The mechanisms suggested to influence metastasis appear to vary with the anaesthetic technique, but generally influence neuroendocrine and immune responses [2]. As a consequence of these findings, numerous, mainly retrospective, trials have been conducted over the years. However, the results of these trials are conflicting, and an expert workshop held in 2014 on this topic [5] concluded that the evidence from retrospective and prospective trials is insufficient to change current practice. This was reinforced by the Cochrane Systematic Review (SR) performed by Cakmakkaya [6] later that year.

Besides the fact that many published studies are retrospective and suffer from confounding, most studies investigate combinations of anaesthetic and analgesic drugs, which makes it a challenge to isolate the contribution of a specific drug. Large-scale RCTs are needed to prove a causal link between anaesthetic techniques and metastasis. Some multicentre trials have been launched [2], and while awaiting their results, we could further evaluate this possible link in animal studies as well.

Animal studies can provide us with information we cannot easily retrieve from human studies: it is much easier, for example, to study the effect of a single anaesthetic technique or drug in an animal study. Furthermore, it is acceptable to provide a placebo treatment to the control group, and combination of drugs can be avoided in animal studies. In addition, the effects of anaesthetic techniques on tumour metastasis can be investigated without conducting surgery, preventing the need for general anaesthesia to be combined with regional anaesthetic techniques.

An evidence-based overview of experimental animal studies would provide us with greater understanding, as a systematic review (SR) and meta-analysis (MA) of all animal studies results in a transparent overview of all available information about, for example, the efficacy of the various anaesthetic techniques in reducing metastases. In addition, it may offer new information that was not available by analyzing each study individually. Another added value of an SR and MA of all animal studies is the new knowledge that can be obtained by evaluating the heterogeneity between the studies. For example, does gender or the type of anaesthetic drug influence the efficacy of the anaesthetic technique on metastasis? Last but not least, SRs of animal studies have previously been used to improve translation of animal research to humans [7].

It needs to be taken into account, however, that various animal models reflect different aspects of the disease, and no animal model represents a perfect match with the clinical situation. To inform clinical practice as optimally as possible, all available evidence from animal studies needs critical evaluation in a systematic review of animal studies. Heterogeneity between studies (e.g. variations between species, environmental conditions and animal models) needs to be explored extensively, as this can provide new (mechanistic) clues for the clinic as well.

This report presents the first SR and MA on the effect of anaesthetic drugs on metastasis in experimental cancer models. This SR provides: 1) a complete and systematic overview of all animal studies on this topic; 2) insight into the efficacy of anaesthetic techniques overall and in subgroups; and 3) an overview of various factors that modify the efficacy in experimental cancer models.

## Methods

This SR investigates the effects of treatment with anaesthetic drugs on number of metastases or metastasis incidence in animals with experimental cancer. The inclusion criteria and method of analysis were specified in advance and documented in a protocol and put online on the SYRCLE website (www.syrcle.nl). As this review was conducted at the same time as a similar review in our department about the effects of a treatment with analgesic drugs on metastasis in experimental cancer [8], parts of the method section overlap.

### Search strategy and paper selection

We searched Medline via the PubMed interface and Embase for original articles concerning *the effects of treatment with analgesic and anaesthetic drugs on metastasis in experimental cancer* on April 21^st^, 2015. In order to design an optimal comprehensive search strategy, we have used SYRCLEs step by step guide [9]. The search strategy involved the following four search components: analgesics, anaesthetics, metastasis and animals [10,11] (for our complete search strategy, see [8]). No language or date restrictions were applied. As we conducted a second review about the effects of analgesic drugs on metastasis in experimental cancer at the same time, the search and first steps of the selection process were combined [8]. When necessary, papers in languages other than English were translated by scientists who were native speakers of that particular language. Reference lists of the selected relevant papers were screened by hand for potentially relevant new papers. No language or data restriction was used. Studies were included in this SR when they met all of the following criteria: 1) the study assessed the effect of an anaesthetic drug currently or historically used in clinical practice on the number or incidence of metastasis in animal models with experimental cancer; 2) the study was performed in animals *in vivo*; 3) the study included an appropriate control group; and 4) the study was an original full paper that presented unique data. Studies were excluded when 1) animals underwent any co-intervention, or 2) animals suffered from co-morbidities.

We used Early Review Organising Software (EROS; Institute of Clinical Effectiveness and Health Policy, Buenos Aires, Argentina) to randomly allocate each reference to two independent reviewers, who screened it for inclusion on the basis of its title and abstract (CH and MB). In case of doubt, the whole publication was evaluated. Full-text copies of all publications eligible for inclusion were subsequently assessed by two independent reviewers (CH and ME) and included when they met our pre-specified inclusion criteria. Disagreement was solved by discussion or by consulting a third investigator (GJS).

### Study characteristics and data extraction

From the included studies, we registered bibliographic data such as authors, year of publication, journal of publication and language. We also extracted data on study design (type of control group), animal model characteristics (animal species, strain, age, weight and sex), cancer model (type of cancer, number of tumour cells, location of injection of tumour cells and type of anaesthetics used to create model), intervention characteristics (type of anaesthetics, route of administration, dose, frequency, timing relative to tumour cell injection, duration of treatment) and outcome measures (either number of metastases or incidence of metastasis, region of metastasis count).

In each of the included publications, we identified all independent comparisons of the number or incidence of metastases in animals with experimental cancer receiving anaesthetic or control treatment. Data on related outcomes, such as number of occupied bones, surface covered with metastases or weight of metastases were excluded. Data on the number or incidence of metastases were extracted when raw data or group averages (mean, median or incidence), standard deviation (SD), standard error (SE) or ranges and number of animals per group (n) were reported or could be recalculated. When outcome measure data were missing, we attempted to contact authors for additional information. When data were only presented graphically, they were measured using Universal Desktop Ruler software (http://avpsoft.com/products/udruler/) by two independent reviewers. When multiple experimental groups were compared to the same control group, the group size of the control group was corrected for the number of comparisons made (n/number of comparisons).

### Assessment of the methodological quality and risk of bias

We used the SYRCLE Risk of Bias tool [12] to assess the risk of bias in the included studies. Two independent reviewers assessed the risk of bias in each included paper (SG, MS, CH, FG) With regard to the risk of attrition bias, we assumed that there had been no exclusion of animals when the number of animals per group mentioned in the materials and methods section was identical to the number stated in the figure legends or results section. A ‘yes’ score indicates low risk of bias; a ‘no’ score indicates high risk of bias; and a ‘?’ score indicates unknown risk of bias.

To overcome the problem of judging too many items as “unclear risk of bias” because reporting of experimental details on animals, methods and materials is very poor [13], we added two items on reporting: reporting of any measure of randomization, reporting of any measure of blinding. For these two items, a ‘yes’ score indicates ‘reported’, and a ‘no’ score indicates ‘unreported’.

### Data synthesis and statistical analyses

Data were analyzed using Comprehensive Meta-Analysis (CMA version2.0). For the outcome measure ‘number of metastases’, the standardized mean difference (SMD) was calculated (the mean of the experimental group minus the mean of the control group divided by the pooled SDs of the two groups). When data were presented as median and percentiles, they were converted to mean and SD. For the outcome measure incidence of metastases, the risk difference (RD) was calculated.

Secondly, the study results were depicted in a forest plot. In case the included comparisons appeared sufficient consistent (overlapping confidence intervals, and no obvious subgroups with other direction of effects) we conducted an overall MA. Individual effect sizes were pooled to obtain an overall SMD and RD and 95% confidence interval. We used the random effects model [14], which takes into account the precision of individual studies and the variation between studies and weighs each study accordingly. When the number of metastasis was measured in multiple regions in the same animals in a particular study, the data were pooled for the overall analyses. For incidence of metastasis we included only those comparisons in the MA that assessed the lung (i.e. a consequence of using the RD). Subgroup analyses were pre-defined in the protocol and put online on the SYRCLE website (www.syrcle.nl) and performed to assess the influence of variables on effect size. The results from subgroup analyses were only interpreted when subgroups contained at least 3 studies or a minimum of 5 comparisons. Subgroup analyses were planned for: anaesthetic technique (general anesthesia, regional or local anesthesia,) type of anaesthetic (volatile, barbiturates, ketamin, propofol, etc), species, sex, region of metastasis, timing and duration of treatment (once, 1, 2, 3, 4 weeks, or more than 4 weeks). We expected the variance to be comparable within the subgroups; therefore, we assumed a common among-study variance across subgroups. For subgroup analyses, we adjusted our significance level according to the conservative Bonferroni method to account for multiple analyses (p* number of comparisons). However, differences between subgroups should be interpreted with caution and should only be used for constructing new hypotheses rather than for drawing final conclusions.

We assessed the possibility of publication bias (when there are more than 10 data points), by visually evaluating the possible asymmetry in the funnel plot for number of metastases, performing Duval and Tweedie's trim and fill analysis and Egger's regression analysis for small study effects. Heterogeneity was assessed using I^2^.

### Sensitivity analyses

In order to assess the robustness of our findings and to further explain observed study heterogeneity, a sensitivity analyses was performed. The impact of 1) single or multiple use of anaesthetic drug treatment; 2) excluding other species than rodents; 3) recalculating median and ranges into means and SDs, and 4) excluding historically used anaesthetics or anaesthetics solely used in developmental countries, was studied.

This systematic review is reported according to the PRISMA statement (S1 PRISMA Checklist) [15].

## Results

### Study selection process

**Fig 1:**
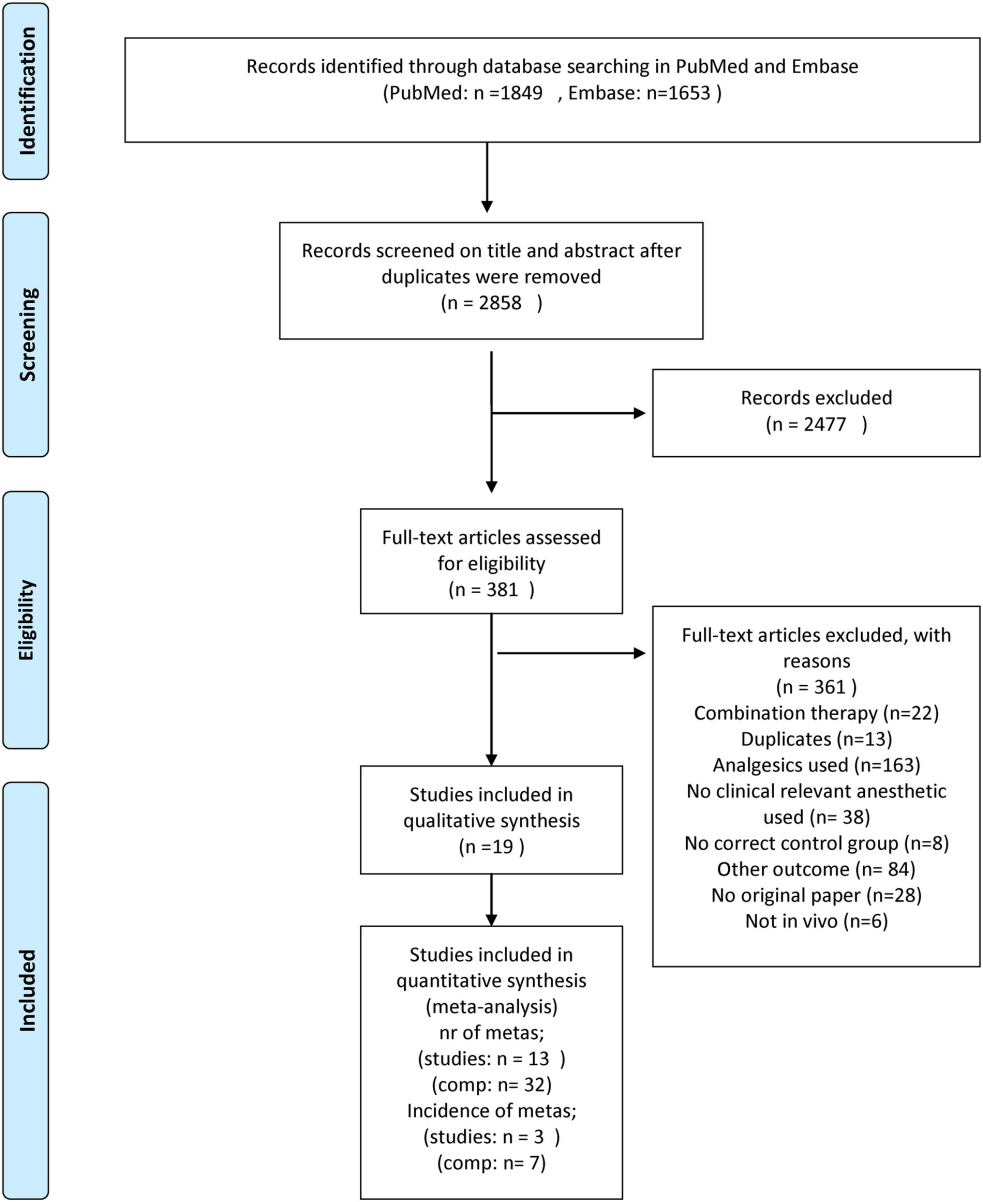
Flow chart of the study selection process. Comp = comparisons (based on the PRISMA flow diagram).

The search strategy in PubMed and Embase yielded 3502 papers. 2858 papers were screened after removal of duplicate citations. Out of these 2858 publications, 19 met our inclusion criteria [16–34]; the remainder was excluded according to the criteria listed in Fig 1. Three of the included papers had to be translated [20,32,33].

### Study characteristics

#### Number of metastases

Data on number of metastases could be retrieved from 15 studies. These 15 studies described 40 independent comparisons. For 9 of these comparisons not all necessary data for meta analysis could be extracted from the papers. Consequently, 3 authors were contacted for additional information. One author responded and these results were included in the meta analysis. Ultimately,32 comparisons could be included in the meta-analyses. Characteristics of all studies and comparisons are listed in S1 Table.

All experiments were conducted in either mice (60%) or rats (40%). Male and female animals were used in 28% and 35% of the comparisons, respectively. Only 2% (1 comparison) used mixed sex groups, and 35% of the comparisons failed to report the sex of the animals used.

The vast majority of studies assessed the number of metastases of skin cancer (30%). Metastases of breast, prostate, blood and bone cancer were studied in 18%, 18%, 10% and 10% of the comparisons, respectively. One study investigated the number of metastases as a consequence of cancer in the lymph nodes. In 13% of the comparisons, it was unclear what type of cancer was studied. Almost all studies investigated the number of metastases in the lungs (80%). 20% assessed the number of metastases in multiple organs.

General anaesthesia was applied in 70% of all comparisons. Either volatile anaesthetics, barbiturates or ketamin was used. Local anaesthesia was administered in 30% of the comparisons (25%). No studies investigating the effects of regional anaesthesia were included.

#### Incidence of metastasis

The incidence of metastases was studied in 3 unique papers. From these 3 papers, data on 11 independent comparisons could be extracted. For four comparisons, which all came from the same original paper (S1 PRISMA Checklist), not all data could be retrieved. Therefore the author was contacted, but no response was obtained. Consequently, these comparisons were not included in the meta analysis. Seven comparisons could be included in the meta-analyses. In 4 out of 11 comparisons (included in the review) rats were used. The other 7 experiments used hamsters. All studies described the sex of the animals. 57% of the experiments were conducted with males. The type of cancer that was used to produce metastasis was unclear in all 3 studies. In all comparisons general anaesthetics were used (barbiturates, ketamin, chloroform or ether).

In 7 out of 11 comparisons the incidence of metastases was determined in multiple regions (lungs, kidney and ganglia). In the other 4 comparisons, solely the incidence of metastasis in the lungs was determined.

### Study quality and risk of bias

**Fig 2:**
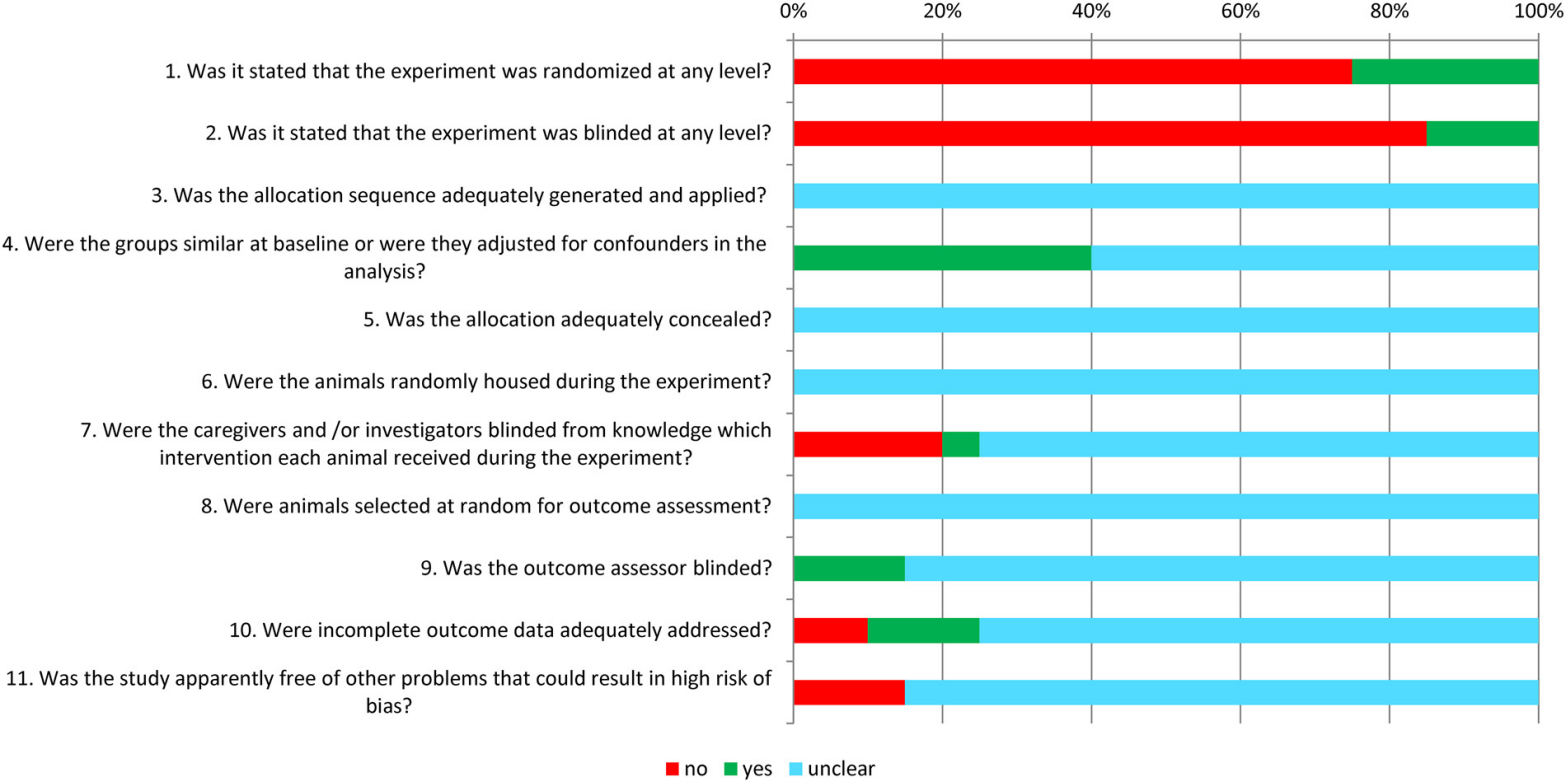
Results of the risk of bias assessment of the 19 studies included in this systematic review. The first two items assess study quality by scoring reporting, a ‘yes’ score indicating reported, and a ‘no’ score indicating unreported. The other items assessed risk of bias, with ‘yes’ indicating low risk of bias, ‘no’ high risk of bias and ‘?’ unclear risk of bias.

Fig 2 shows the overall results of our risk of bias assessment of the 19 studies included in this SR (papers on number and incidence of metastases). Because reporting of experimental details on animals, methods and materials is generally poor [13], and this will lead to many judgements of an unclear risk of bias, we decided to score two items on reporting as well: reporting of any measure of randomization and reporting of any measure of blinding.

Only 25% of the papers mentioned the use of randomization at any level. Blinding of the experiment at any level was only mentioned in 15% of the studies. The risk of bias assessment revealed that it was the outcome assessor who was blinded in those studies. For the other risk of bias items, poor reporting led to an unclear risk of bias in the majority of assessments. For example, none of the authors described the allocation sequence or whether or not this sequence had been concealed. None of the papers gave details on random housing, and as a consequence all studies had to be scored as an unclear risk of bias.

In 2 studies there were other sources that caused a high risk of bias: the control treatment procedure was not identical to the procedure of the experimental group in both these studies.

### Meta-analysis of the efficacy of treatment with anaesthetic drugs

#### Number of metastases; efficacy of anaesthetic drugs

Regarding the outcome number of metastases, 13 studies describing 32 independent comparisons could be included in MA. Plotting the standardized mean differences of the individual comparisons immediately revealed large variation between the studies (in both magnitude and direction of effect). Exploring the possible cause of variation, with help of the characteristics table (S1 Fig), revealed that all studies with extreme outcomes had had the same unique feature. They all investigated that effect of local anaesthetics on tumour metastases. Therefore we decided not to pool all anaesthetic techniques, but to separate general from local anaesthetics.

**Fig 3:**
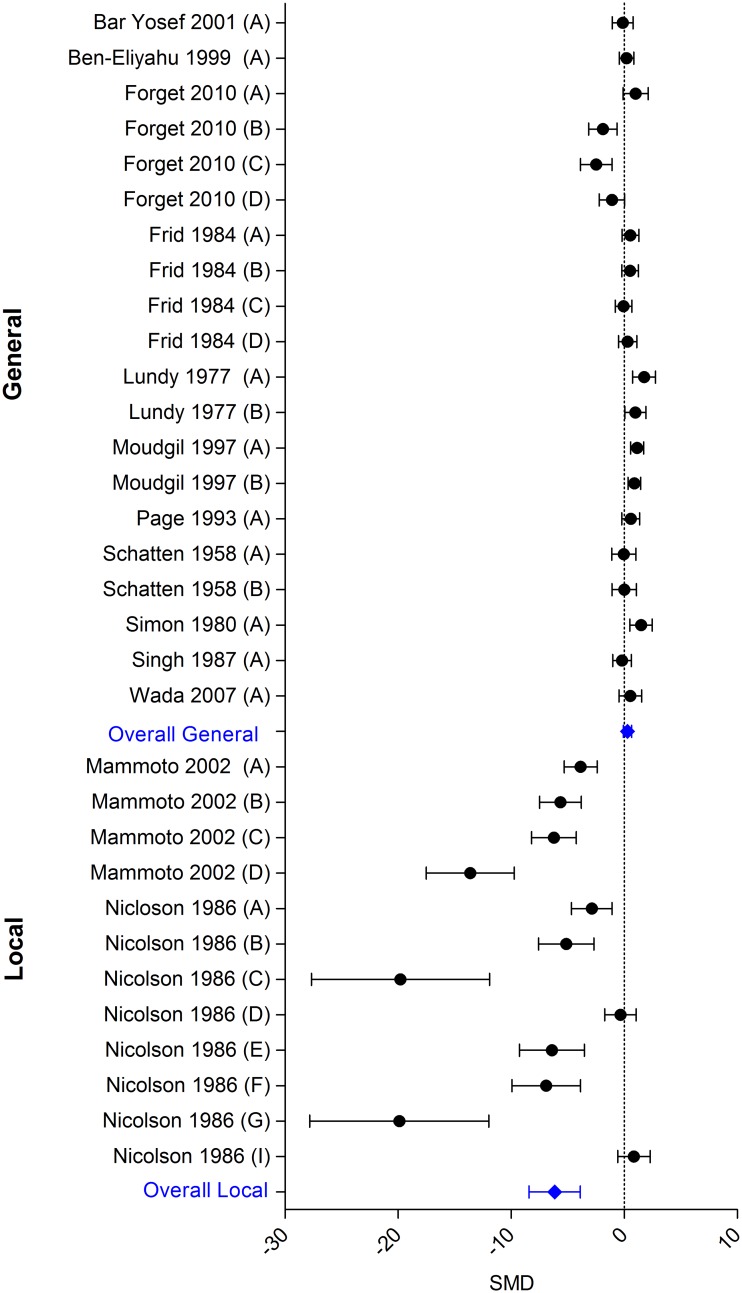
Forest plot of the impact of general and local anaesthetics on the number of metastases. Left side: reduced number of metastases; right side: increased number of metastases. An overall beneficial effect of local anaesthetics was observed. General anaesthetics do not have an effect. Data presented as SMD and 95% CI.

Eleven studies, including 20 independent comparisons, studied the effect of general anaesthetics on the number of metastasis in experimental cancer. Two studies, containing 12 independent comparisons, focused on local anaesthetic techniques. In both of these studies the local anaesthetic drugs were administered locally (Mammoto et al. administered lidocaine subcutaneously near the tumour [23], whereas in the study of Nicolson, the injected cancer cells were pretreated with tetracaine [25]). The overall analysis of general anaesthetics (Fig 3) showed no effect (SMD 0.29 [-0.07; 0.65]). In contrast, local anaesthetics (locally administered) appeared to significantly reduce the number of metastases (SMD -6.15 [-8.42; -3.88]). Between-study heterogeneity was high for both general and local anaesthetics (I^2^ 73.8% respectively 92.0%).

#### Number of metastases; effects of study characteristics on efficacy of anaesthetic drugs

The results from subgroup analyses were only interpreted when subgroups contained at least 3 studies or a minimum of 5 comparisons. As a consequence no subgroup analyses for local anaesthetics have been conducted.

**Fig 4:**
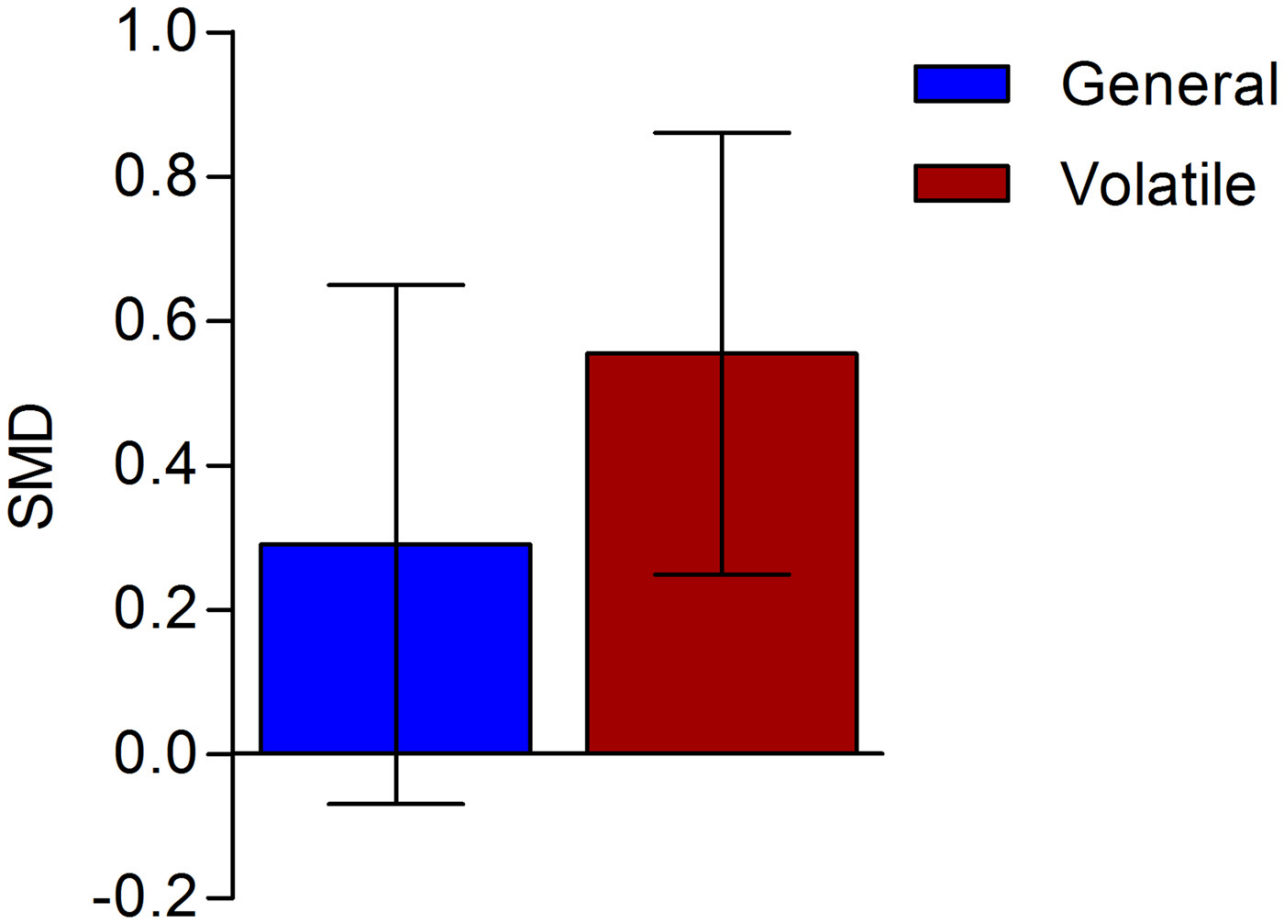
The impact of volatile anaesthetics. The columns depict the effect estimate (SMD) with the 95% confidence interval. The results from subgroup analyses (volatile anaesthetics) were only interpreted when subgroups contained at least 3 studies or 5 independent comparisons

The type of general anaesthetic used, appears to influence the results. Although the overall analysis of all general anaesthetics showed no effect on the number of tumours, volatile anaesthetics significantly increased the number of metastases in experimental cancer (Fig 4; SMD 0.54 [0.24; 0.84] n = 11; I^2^ = 42%.). In 5 of these comparisons, halothane (which is widely used in developing countries) was used. In two comparisons, either isoflurane or sevoflurane were used, and the other comparisons used historic anaesthetics (chloroform (n = 1), ether (n = 2), methoxyflurane (n = 1))). The subgroups of the remaining comparisons (barbiturates and ketamin) were too small to conduct reliable assessments (respectively n = 3 and n = 4).

Within the subgroup of volatile anaesthetics no statistically significant differences were observed between the various species (mice and rats). Other subgroup differences could not be investigated as the subgroups contained too few comparisons.

#### Incidence of metastasis; efficacy of anaesthetic drugs

Regarding our second outcome, incidence of metastasis, only the effect of general anaesthetics could be investigated, since no studies assessing the effect of local anaesthetics on metastasis incidence were identified.

**Fig 5:**
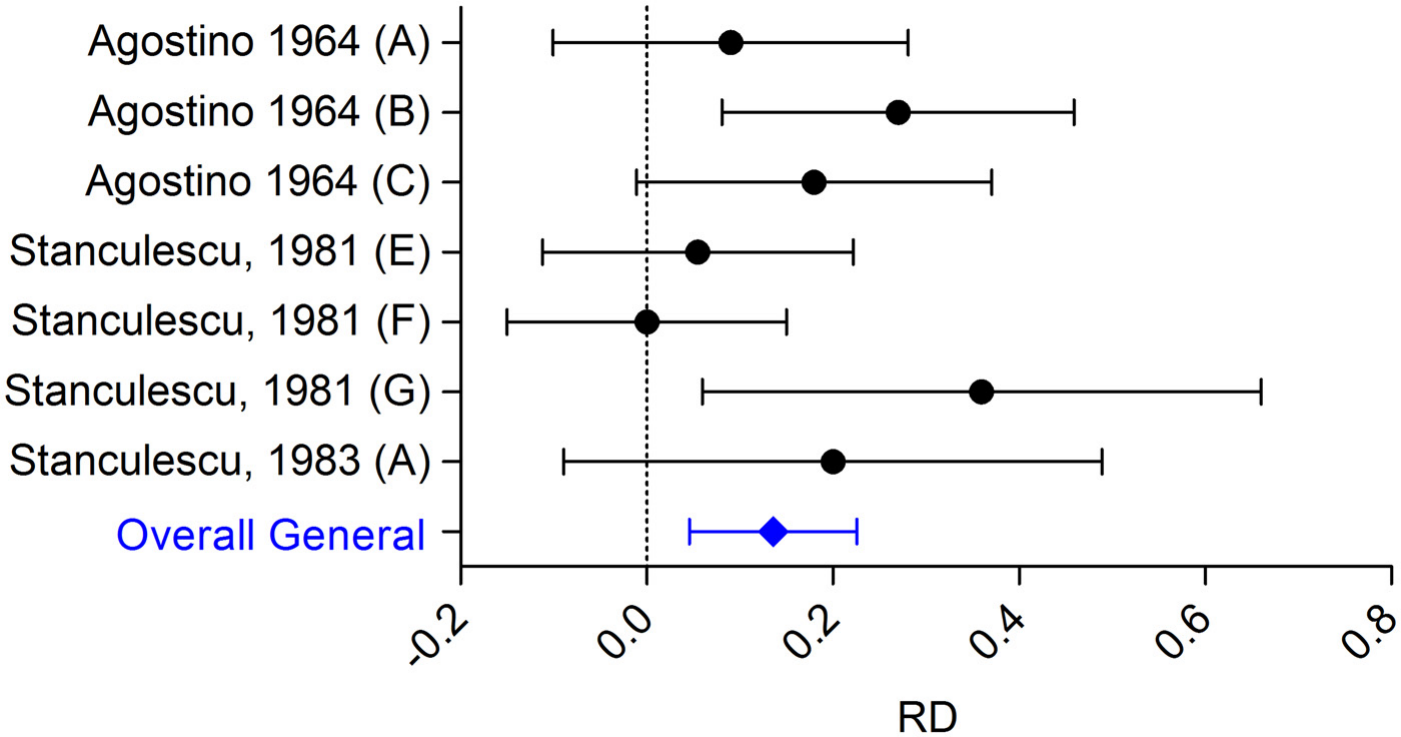
Forest plot of the impact of general anaesthetics on the incidence of metastases. Left side is reduced incidence of metastases, right side equals increased incidence of metastases. General anaesthetics increase the incidence of metastases. Data are presented as Risk Difference (RD) and 95% CI.

Three studies, describing 7 comparisons could be included in the MA. Overall analysis (Fig 5) showed that general anaesthetics increase the incidence of metastasis (RD: 0.136 [0.045, 0.226]; n = 7, I^2^ 31%,) as the volatile anaesthetic did for the outcome number of metastasis.

#### Incidence of metastasis; effects of study characteristics on efficacy of anaesthetic drugs

Within the general anaesthetic group, 2 comparisons investigated the effects of volatile anaesthetics, 1 studied the effects of ketamin and 4 the effects of barbiturates. However, the subgroups were too small to draw reliable conclusions.

#### Sensitivity analysis

To assess the robustness of our findings and to further explain the observed study heterogeneity, we performed a sensitivity analysis for some of the decisions we made in the inclusion and exclusion criteria. Our analysis showed that excluding the studies that applied surgery during the experiment resulted in a significantly increased number of metastases in the general anaesthesia group. The other sensitivity indicators did not affect the results.

In order to verify whether the conclusion that volatile anaesthetics increase the number of tumours is the consequence of the studies using historically used anaesthetics or anaesthetics only used in developmental countries, we conducted additional analyses in which the studies using ether, methoxyflurane, chloroform and halothane were excluded. The conclusion that volatile anaesthetics increase the number of tumours did not change, and therefore appears robust (excluding historically used anaesthetics; SMD 0.66 [0.237–1.075], n = 7; excluding historically used anaesthetics and halothane SMD 0.81 [0.32–1.29], n = 2).

#### Publication bias

The possible presence of publication bias was assessed for the effects of local and general anaesthetics on the number of metastases. Inspection of both funnel plots suggested some asymmetry due to an underrepresentation of studies with moderate precision and increased number of metastases as a consequence of treatment with anaesthetics. Duval and Tweedie's trimm and fill analysis resulted in 5 extra data points for local anaesthetics, and 4 for general anaesthetics, indicating the presence of little publication bias and a small underestimation of the summary effect size (S1 Fig).

## Discussion

Whether anaesthetic drugs and techniques influence tumour recurrence and metastases is a matter of strong debate. It has been hypothesized, for example, that regional anaesthesia techniques and local anaesthetics decrease tumour metastasis [3,35], but a recent Cochrane SR clearly illustrates that there is a lack of evidence for an effect of regional anaesthesia techniques on long-term outcomes after cancer surgery [6]. In addition, it has been suggested that volatile anaesthetics influence tumour recurrence and metastasis as well, as they appear to have immunosuppressive effects [4]. However, no RCTs have currently been published on the effects of volatile anaesthetics on tumour metastasis in humans.

While awaiting the results of ongoing RCTs regarding this topic in patients, we have analyzed the evidence regarding the influence of anaesthetic drugs and techniques on tumour metastasis in animal studies. Animal studies can provide us with information that we cannot easily retrieve from human studies. It is much easier, for example, to isolate the effect of specific anaesthetic drugs in animal models, as the effects of anaesthetic techniques on tumour metastasis can be investigated without conducting surgery.

Our SR and MA showed that anaesthetics influence the number and incidence of metastases in experimental cancer models. Locally administered local anaesthetics appear to decrease the number of metastases, whereas general anaesthetics, and especially volatile anaesthetics, appear to increase the number and risk of metastases in experimental cancer models.

The finding that locally administered local anaesthetics appear to decrease the number of metastases in animals is very promising but should be further investigated for two main reasons. First of all, it is especially interesting to investigate whether systemically administered local anaesthetics diminish tumour metastases as well, as perioperative lidocaine infusion has been introduced in recent years to improve pain management after major surgery [6]. Secondly, the reliability of our finding is highly dependent on the number and quality of the studies being analysed. For the effects of locally administered local anaesthetics on metastasis only two studies (with 12 comparisons) were identified, and these two studies varied considerably in their design. Mammoto et al., for example, administered lidocaine subcutaneously [23], whereas the injected cancer cells were pretreated with tetracaine in the study by Nicolson [25]. In addition, the methodological quality of the included studies was difficult to assess due to poor reporting of many essential details. It is recommended, therefore, to further investigate the potentially beneficial effect of various local anaesthetics in experimental cancer models.

It has been hypothesized that local anaesthetics influence metastasis by inhibiting proliferation and migration of cancer cells and inducing apoptosis [4]. Another hypothesis could be that local anaesthetics, as has been suggested for regional anaesthetics [6], are not that effective in themselves, but that their effect is a consequence of decreasing opioid administration. The latter hypothesis is not confirmed by this review as we observed an effect of locally administered local anaesthetics as such (e.g., local anaesthetics versus vehicle). In addition, a previous SR by our laboratory suggests that opioids do not influence tumour metastasis at all [36].

Volatile anaesthetics appear to have immunosuppressive effects [2,4] and up-regulate cancer cell processes such as angiogenesis and proliferation [2]. These findings offer a possible mechanistic explanation for the results observed in our SR showing that volatile anaesthetics increase the risk of tumour metastasis in experimental cancer. However, the majority of comparisons included in our MA used either halothane (an anaesthetic widely used in developing countries, but not so much in western countries) or historically used anaesthetics. Nevertheless, sensitivity analysis showed that, after excluding the studies using either the historically used anaesthetics or halothane, the number of tumours remained significantly increased. Taken together, these observations that volatile anaesthetics seem prone to increase the number of metastases appears robust, but warrants continued and more extensive investigation. More preclinical studies on the influence of modern volatile anaesthetics affecting metastases are needed, also to strengthen these findings. However, a very recent clinical study already showed an association between volatile inhalational anaesthesia and a reduction in long term survival of cancer patients [37]

A major and urgent priority for future research are studies investigating the effect of either regional anaesthesia or total intravenous anaesthesia (TIVA) on tumour metastasis in experimental cancer. We did not identify any experiments investigating the effect of regional anaesthesia that fitted our inclusion criteria. Some experiments investigated the effect of spinal block, but they could not be included in this review as they combined treatment of both anaesthetics and analgesics (e.g., combination therapy) [17,34]. In addition, none of the included studies investigated the effect of Propofol, which is surprising as this drug is extensively used in clinical practice.

### Limitations

An important limitation of this review is the poor evidence base. Firstly, the methodological details in many of the animal studies included in our review were poorly reported. This seriously hampers reliable risk of bias assessment and makes it harder to draw reliable conclusions from the included animal studies. Poorly reported studies were included in this review, because papers that do not report essential details, are not necessarily methodologically impaired. Nevertheless, it is important to stress that there is a great need for more consistent reporting of essential details regarding experimental design for future animal experiments.

Secondly, for many analyses the number of studies was low and the variation between the studies high. This influences the reliability of the conclusions drawn from this systematic review.

In case the number of primary studies studying the effect of anaesthetics on tumour metastasis increases, and thus subgroups investigating the effects of many important study characteristics increase as well, the observed between-study heterogeneity can be explored more extensively. This can help to inform the design of future clinical trials and, ultimately, clinical practice. For now, we have accounted for anticipated heterogeneity by using a random rather than fixed effects MA. In addition, we conducted multiple sensitivity analyses to increase our confidence in the results. We have shown that some of the possible sources of heterogeneity, such as intravenous or local inoculation of tumour cells or type of cancer induced, did not alter the conclusions.

It is important to stress that a large variation between the included animal studies is not problematic per se. In case of a sufficient number of studies, the added value of many meta-analyses of animal studies is often the new knowledge that can be obtained by evaluating the heterogeneity between the studies.

Furthermore, it is important to realize that the various animal models included in this review reflect different aspects of the disease, and no animal model is a perfect match to represent the full clinical situation. When the results of a review like this will be used to inform the clinical field, the indirectness of the results need to be taken into account.

Thirdly, the certainty in the observed effects is also decreased as a consequence of the suggested presence of publication bias. We have identified some funnel plot asymmetry, and the Duval and Tweedie's trim and fill analysis would predict a small underestimation of the summary effect size. However, the interpretation of the results of a funnel plot using SMDs may be somewhat unclear and is a topic for further research.

### Clinical relevance and recommendations

The results of our review are broadly consistent with clinical reports that anaesthetic technique does seem to affect cancer outcomes. It therefore supports the need for clinical research to prove a possible causal link between anaesthetic techniques and metastases.

Although more research is necessary, there is no indication based on the available evidence from animal studies to suggest that locally administered local anaesthetics during surgery in cancer patients could be harmful. It might even be beneficial. Volatile anaesthetics, however, might increase metastasis and clinical trials investigating this possibly harmful effect should get research priority.

It is important to exercise great caution in translating the results of animal studies to the clinical situation. No animal model is a perfect match to represent the clinical situation. Many species, for example, metabolize drugs differently as compared to humans. Comparable results in multiple species would increase our confidence in the results and applicability for patients. Ideally, randomized controlled trials with cancer patients receiving either an anaesthetic or placebo treatment are needed to confirm the results of our review. Such trials of course will never meet ethical approval, therefore, future RCTs should focus on comparing one anaesthetic technique against another.

New animal studies are also warranted and should, based on the results of this review, initially focus on the effects of regional anaesthetics, and the most commonly used volatile and local anaesthetics on tumour metastasis. Interestingly, intravenous administration of local anaesthetics have gained much attention for post operative pain treatment. Therefore, the effect of intravenous administration of local anaesthetics on tumour metastasis requires further research. Last but not least, there appears to be a great lack of animal data regarding the use of propofol. Given the extensive use of this drug in worldwide clinical practice for induction and maintenance of anaesthesia, ongoing sedation in critical care areas and the increasingly popular technique of total intravenous anaesthesia (TIVA), lack of animal data here is a significant shortcoming of the current evidence base and should therefore be a major and urgent priority for future research.

It also needs to be unravelled if efficacy varies between varies types of cancer and various species. Only two rodent species were used in most of the analyses in this review. Consistency in the observed finding in multiple species will increase our confidence in the effect and improve translation of results to the clinical setting.

However, filling up the above mentioned gaps of knowledge is only of true value when scientists and editorial boards embrace correct reporting and conduct guidelines for experimental animal research [12,38,39].

## Supporting Information

S1 Fig Assessment of publication bias. Inspection of the funnel plot suggests an underrepresentation of studies with moderate precision and an increased risk of metastasis as a consequence of treatment with analgesics in animals with experimental cancer. Open circles represent the observed data, closed circles the estimations of the missing data.
(TIF)

S1 PRISMA Checklist (DOC)

S1 Table Characteristics of all studies and comparisons. Summary of all publications included in the systematic review. Multiple comparisons can be extracted from one publication.? = unclear/ unreported. F = female, m = male, G = General anaesthetics, L = local anaesthetics, iv = intravenous, id = intradermal, ip = intraperitoneal. Sc = subcuteaneous, w = weeks, d = days, incidence = incidence of metastases, number = number of metastases.
(XLSX)
