# Supplementary figures and images for: A Systematic Review of the Modifying Effect of Anaesthetic Drugs on Metastasis in Animal Models for Cancer
Source: PLoS One. 2016 May 26;11(5):e0156152. doi: 10.1371/journal.pone.0156152 (PMC4882001; doi:10.1371/journal.pone.0156152)

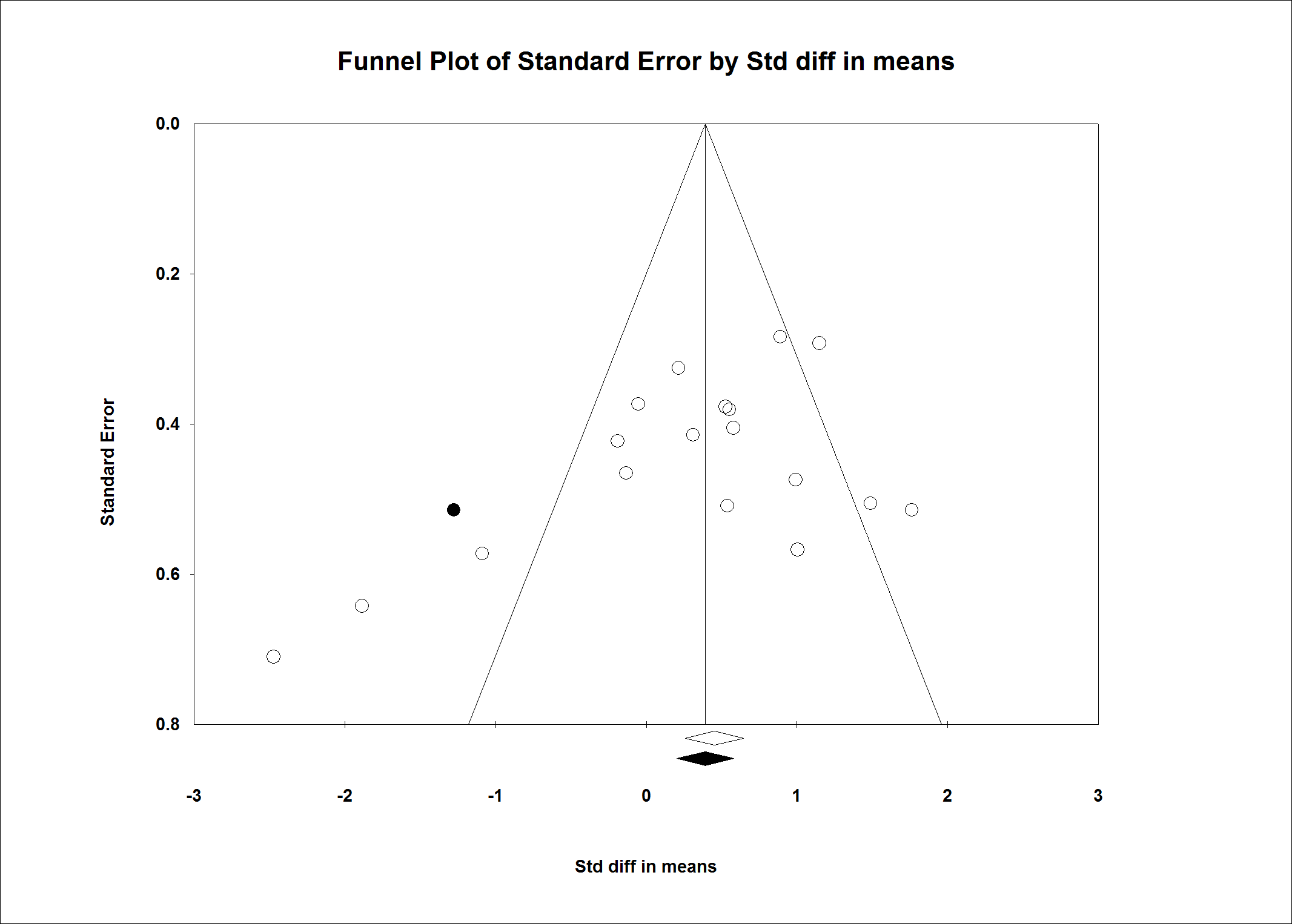

Supplement: S1 Fig — Inspection of the funnel plot suggests an underrepresentation of studies with moderate precision and an increased risk of metastasis as a consequence of treatment with analgesics in animals with experimental cancer. Open circles represent the observed data, closed circles the estimations of the missing data. (TIF) [file pone.0156152.s001.tif]
